# Effect of Nutrition on Age-Related Metabolic Markers and the Gut Microbiota in Cats

**DOI:** 10.3390/microorganisms9122430

**Published:** 2021-11-25

**Authors:** Eden Ephraim, Dennis E. Jewell

**Affiliations:** 1 Pet Nutrition Center, Hill’s Pet Nutrition, Inc., Topeka, KS 66617, USA; 2 Department of Grain Science and Industry, Kansas State University, Manhattan, KS 66506, USA; djewell@ksu.edu

**Keywords:** cats, aging, plasma, feces, metabolome, microbiota

## Abstract

Age-related changes in the gut microbiota and metabolites are associated with the increased risk of detrimental conditions also seen with age. This study evaluated whether a test food with potential anti-aging benefits results in favorable changes in plasma and fecal metabolites and the fecal microbiota in senior cats. Forty healthy domestic cats aged 8.3–13.5 years were fed a washout food for 30 days, then control or test food for 30 days. After another 30-day washout, cats were switched to the other study food for 30 days. Assessment of plasma and fecal metabolites showed lower levels of metabolites associated with detrimental processes (e.g., uremic toxins) and higher levels of metabolites associated with beneficial processes (e.g., tocopherols) after cats consumed the test food compared with the control food. A shift toward proteolysis with the control food is supported by higher levels of amino acid metabolites and lower levels of carbohydrate metabolites. Operational taxonomic units of greater abundance with the test food positively correlated with carbohydrate and nicotinic acid metabolites, and negatively correlated with uremic toxins, amino acid metabolism, secondary bile salts, and branched-chain fatty acids. Taken together, the test food appears to result in greater levels of metabolites and microbiota associated with a healthier state.

## 1. Introduction

Research into the microbiota of humans and animals has expanded at a rapid pace over the last several years. Dietary factors direct the composition and metabolic output of the gastrointestinal microbiota, which in turn influences the health of the host through the production of postbiotics [1]. Aging has a profound effect on the composition and diversity of the gastrointestinal microbiota [2], as has been shown in humans [3,4] and dogs [5,6]. Prior work has characterized the feline fecal microbiota at various stages of life, and also demonstrated shifts in its composition with age and food [7,8,9].

Age-related changes in the gut microbiota may be associated with immunosenescence, or age-related dysfunction of the immune system, in humans [3]. Immunosenescence may lead to “inflammaging,” low-grade chronic inflammation that contributes to age-related diseases [10]. Indeed, detrimental effects of aging in cats include an increased risk of a variety of conditions associated with inflammation such as chronic kidney disease (CKD), diabetes, gastrointestinal disease, cancer, and cognitive decline [11,12].

With an average life expectancy of about 12 years for pet cats [13] and consideration of cats > 10 years of age as senior [12], it is of great interest to investigate foods that mitigate the adverse effects of aging. Previous research on foods with a similar formulation to the one tested here [14] showed benefits in kidney parameters in senior cats, including increased glomerular filtration rate (GFR); decreased serum levels of symmetric dimethylarginine (SDMA), a biomarker for early CKD [15]; and lower serum levels of 3-indoxyl sulfate, a uremic toxin. The goal of this study was to evaluate the effect of a test food with potential anti-aging benefits on plasma and fecal metabolites and the gut microbial composition of senior cats.

## 2. Materials and Methods

### 2.1. Animals, Study Foods, and Experimental Design

Forty domestic shorthair cats between 8–16 years of age, owned by Hill’s Pet Nutrition, Inc., all spayed or neutered, were included in this study. Those with chronic disease conditions such as inflammatory bowel disease, dermatitis, food allergy, cancer/tumor, kidney disease, liver disease, or chronic urinary tract infections were excluded. Cats were to be removed from the study if they lost more than 15% of their body weight or had a low intake that could result in that weight loss. All cats were individually housed at the Hill’s Pet Nutrition Center and were provided with regular opportunities to exercise and socialize with other cats. The Hill’s Institutional Animal Care and Use Committee (permit CP632) and Animal Welfare Committee approved this study protocol in accordance with the National Research Council guide [16].

The ingredient preponderance for the control food was wheat, corn gluten meal, rice, pork fat, chicken, egg, beet pulp, flax seed, wheat gluten, fish oil, carnitine, soybean oil, and oat fiber. For the test food, the preponderance was brown rice, corn gluten meal, pea, chicken, oat groats, fiber blend (broccoli and tomato pomace), soybean oil, beet pulp, fish oil, and carnitine. Oat groats were added to the test food to serve as a source of beta-glucan that could reach the colon and be fermented by saccharolytic microbes. The fiber blend of broccoli and tomato pomace was to serve as a source of natural antioxidant polyphenols known to have a protective effect against age-associated cognitive decline [17,18]. Both foods were supplemented with vitamins, minerals, and palatability enhancers. The amount of fish oil in the control food was almost double the amount in the test food. The washout food contained similar ingredients to the control food but did not contain beet pulp, oat groats, or the fiber blend, so that the effects of those ingredients would be observable. Food analytical measurements were determined using Association of Analytical Communities methods by Eurofins Scientific Inc. (Des Moines, IA, USA). Digestibility assays were performed as previously described [19].

**Figure 1:**
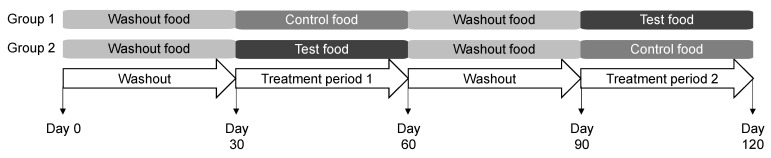
Study design in which cats consumed the washout, control, and test foods.

After a 30-day period during which all cats consumed the washout food, cats were split into two equal groups in which one group consumed the control food and the other the test food for 30 days (Figure 1). Next, all 40 cats were fed washout food for 30 days before they were switched to the other food for 30 days. This crossover design controls for the time effect and also allows each cat to serve as its own control, especially important in studies of microbiota to account for inter-individual variation [20]. The washout period between each study food was to eliminate a carryover effect from the food consumed in the first treatment feeding period [21]. Cats had unlimited access to water throughout the day. All cats were offered fresh food with amounts available for consumption to maintain body weight. Daily food intake was recorded. Blood and fecal samples were collected at the end of each 30-day period for analyses. Blood chemistry was analyzed as described by Hall et al. [15].

### 2.2. Stool Collection, Scoring, and Sample Processing

On stool collection days, caretakers inspected the litter boxes every 15 min for stool production. Upon observation of a stool, it was scored on a scale ranging from 1 (not solid, > 75% liquid) to 5 (cylindrical, > 80% firm) as previously described [22]. Fresh fecal samples were homogenized (Thinky Mixer, Thinky USA Inc., Laguna Hills, CA, USA), and were frozen as aliquots at −80 °C as previously described [23].

### 2.3. Metabolite Analysis

Plasma and fecal metabolites were analyzed by Metabolon, Inc. (Morrisville, NC, USA). SCFAs were extracted, acidified with methyl-t-butyl ether, and resolved by capillary gas chromatography as previously described [24]. Maximum and minimum detection levels were used for fecal SCFA measurements below or above the detection limits.

### 2.4. Bioinformatics Processing

Microbiome analysis of frozen fecal samples was conducted as described by Hall et al. [25]. The Qiagen MagAttract Power Microbiome DNA/RNA EP DNA isolation kit (Qiagen Cat. No. ID:27500–4-EP; Germantown, MD, USA), optimized for use with the Eppendorf epMotion 5075 TMX platform (Eppendorf, AG, Hamburg, Germany), was used for total DNA extraction. PCR amplification spanned the V3–V4 hypervariable regions of the 16S rRNA gene. Amplicon sequencing was performed using the Illumina library preparation protocol (15044223 Rev. A); sequences were de-multiplexed to obtain FASTQ Files, and bacterial taxonomic classification was per the GreenGenes reference taxonomy. Centered log-ratio (CLR) transformation of the copy-corrected operational taxonomic units (OTU) count data was performed to enable appropriate statistical analysis.

### 2.5. Statistical Analysis

Data were analyzed by treatment food, and data for both groups were combined as appropriate for the control and test foods. Statistical analyses were performed in JMP, version Pro 15 (SAS Institute, Cary, NC, USA). Metabolomics data were log-transformed prior to matched-pair analysis, performed to test whether means were different between treatments. Linear regression analyses are reported by the square of Pearson’s correlation coefficient (*r*^2^) and *p*-values. Log transformation was applied to non-normally distributed variables. Statistical significance was established with *p* ≤ 0.05 and FDR-corrected *p* ≤ 0.1.

## 3. Results

### 3.1. Food, Study Design, and Animals

The major differences between the control and test foods were that the test food contained brown rice as well as a fiber blend of broccoli and tomato pomace. Fish oil levels in the control food were almost twice that of the test food. The control and test foods had similar levels of ash, crude fiber, and crude protein as seen via proximate analyses (Table 1). Higher levels of crude fat, moisture, and omega-3 fatty acids were observed in the control food compared with the test food.

Digestibility parameters were similar between the two foods, with slightly higher apparent and true protein digestibility in the test food (Table 2). Apparent fiber digestibility was about twice as high in the control food as in the test food. Only true protein digestibility was significantly different between the foods (*p* = 0.001).

Cats were divided into two groups of 20, with Group 1 receiving the control food during the first 30-day feeding period and the test food during the second 30-day feeding period, and Group 2 receiving the foods in the reverse order, with a 30-day washout between the feeding periods. The mean ± standard deviation age at the start of the study was 11.5 ± 1.6 years in Group 1 (7 males, 13 females) and 11.3 ± 1.6 years in Group 2 (6 males, 14 females). No adverse events were encountered during the study period, and no cats needed to be removed from the study.

Body weights increased following consumption of the control and test foods. However, these increases were not significantly different between the two foods, despite a significantly higher daily intake with the test food (Table 3). The average stool score was slighter higher, indicating greater firmness, in the control food group compared with the test food group, though both were in the acceptable range.

### 3.2. Effect of the Control and Test Foods on Blood Chemistry and Plasma Metabolites in Senior Cats

Following consumption of the study foods, blood concentrations of both creatinine and blood urea nitrogen (BUN) were significantly higher when cats had consumed the control food compared with the test food (Table 4).

Several significant differences in plasma metabolites of interest were observed between the two food types (Table 5; Supplemental Table S1). A number of uremic toxins, including 3-indoxyl sulfate, 5-hydroxyindole sulfate, 6-hydroxyindole sulfate, 3-hydroxyhippurate, 3-hydroxyphenylacetate sulfate, urea, dimethylarginine (SDMA + ADMA), 1-methylguanidine, 4-ethylphenyl sulfate, and 2-oxindole-3-acetate were higher with the control food. The advanced glycation end product (AGE) pyrraline and several gamma-glutamyl amino acids were also found at greater plasma levels following consumption of the control food. In contrast, tocopherols, which are antioxidant markers, and some products of nicotinate and nicotinamide metabolism, were higher in the test food.

### 3.3. Effect of Control and Test Foods on Fecal Metabolites in Senior Cats

Similar to the plasma metabolites, higher levels of uremic toxins and lower levels of tocopherols and products of nicotinate and nicotinamide metabolism were observed in feces after cats consumed the control food compared with the test food (Table 6; Supplemental Table S2). Generally, levels of amino acid metabolites were higher and levels of carbohydrate metabolites in feces were lower with the control food. In addition, several secondary cholate-derivative bile salts increased with the control food, while the primary bile salt cholate decreased.

Fecal SCFA analysis showed significantly higher levels of butyric acid and lower levels of propionic acid with the test food compared with the control food (Table 7). Levels of the BCFAs isobutyric acid and isovaleric acid were significantly higher with the control food.

### 3.4. Effect of Control and Test Foods on Fecal Microbiota in Senior Cats

**Figure 2:**
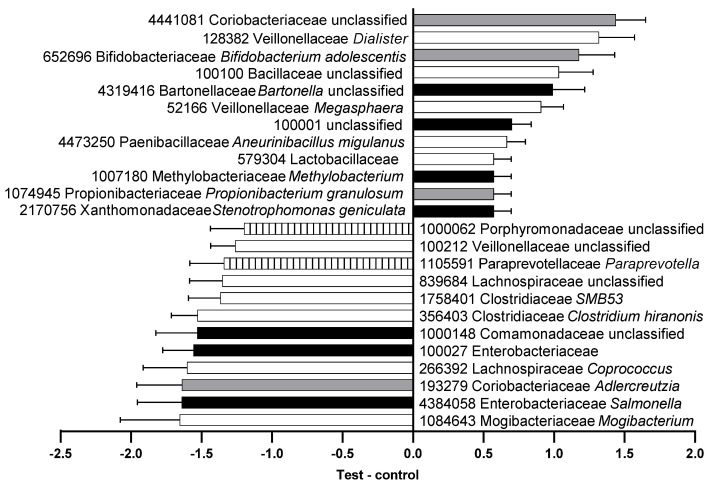
Relative abundance (center log-ratio) of the difference between feces from the test and control food groups in operational taxonomic units (OTU) that were significantly different. OTU number, family, and genus are shown with species where available. Black, phylum Proteobacteria; gray, phylum Actinobacteria; white, phylum Firmicutes; striped, phylum Bacteroidetes.

Seventy-seven OTUs significantly differed in feces from cats fed the control versus test foods, 26 of which were more abundant following consumption of the control food and 51 more abundant after the test food (Supplemental Table S3). Several families of saccharolytic bacteria such as Coriobacteriaceae, Veillonellaceae, Bifidobacteriaceae, and Lactobacillaceae were more abundant after cats consumed the test food (Figure 2).

### 3.5. Correlations among Plasma and Fecal Metabolites and OTUs

Several plasma indoles correlated with OTUs that were significantly different between the control and test foods (Table 8; Supplemental Table S4). Of note, the OTUs that were of greater abundance after cats consumed the control food positively correlated with these uremic toxins, while those of greater abundance with the test food were negatively correlated.

In correlation analyses of fecal metabolites with OTUs, the indolic uremic toxins positively correlated with OTUs that were increased with the control food and negatively correlated with those that were increased with the test food (Table 9), similar to the observation with the plasma metabolites. OTUs that were of higher abundance with the test food were positively correlated with the primary bile salt cholate but negatively correlated with the secondary bile salts. In addition, OTUs that were increased with the test food were positively correlated with carbohydrate metabolites and negatively correlated with dipeptides, while the reverse was observed with the OTUs of greater abundance with the control food (Supplemental Table S4). Positive correlations with several metabolites of nicotinate and nicotinamide, such as NAD^+^, NaMN, and trigonelline, were observed with the OTUs of greater abundance after consumption of the test food (Supplemental Table S4).

In correlation analyses of fecal SCFAs and OTUs, positive correlations were observed with acetic acid and butyric acid for the OTUs that were of greater abundance following consumption of the test food, and negative correlations with propionic acid, isobutyric acid, and isovaleric acid (Table 10). The opposite trend was seen with SCFAs and OTUs that were higher with the control food.

## 4. Discussion

In this study, lower levels of metabolites associated with detrimental processes and higher levels of metabolites associated with beneficial processes were seen after senior cats consumed the test food compared with the control food. Levels of amino acid metabolites were higher and levels of carbohydrate metabolites in feces were lower with the control food, indicating a possible shift in metabolism. A relatively small number of OTUs differed in the feces from cats fed the control and test foods. Generally, much greater differences in gut microbiota have been observed when comparing disease states with healthy controls than when comparing consumption of different food types in humans or animals [9]. Despite these relatively small differences between food types in the present study, many of the OTUs of greater abundance with the test food have been associated with healthier states, particularly in prior studies focused on CKD or aging. Notably, OTUs of greater abundance with the test food were positively correlated with carbohydrate metabolites, several nicotinic acid metabolites, and were negatively correlated with uremic toxins, amino acid metabolism, secondary bile salts, and BCFAs. The opposite effects were observed with the OTUs that were greater following consumption of the control food.

Digestibility parameters were similar in both study foods and to those from another study in cats [26], though nutrient digestibility decreases with old age in cats [27]. The apparent fiber digestibility was about twice as high in the control food compared with the test food, which is likely due to the presence of brown rice, known to have low digestibility, in the test food. The significantly higher true protein digestibility with the test food may have contributed to the observed benefits.

A number of metabolites associated with renal dysfunction were lower in plasma and/or feces from cats fed the test food compared with the control food in this study. This is of particular interest since reduced kidney function with age has been observed in cats as measured by GFR [28]. Increased inflammation likely greatly contributes to this, as both oxidative stress and inflammation are greater with normal aging and in CKD [29].

Tryptophan is converted by gut microbes to indole, which then enters circulation and is converted into indoxyl sulfate by the liver [30]. Indoxyl sulfate is normally cleared by the kidneys but is increased in the circulation in those with CKD and is an indicator of decreased renal function in humans and companion animals. Indoxyl sulfate negatively correlated with estimated GFR in people with early renal function decline [31], suggesting that it may contribute to further decline [32]. In addition, cats with stage 2–4 CKD had significantly higher levels of serum indoxyl sulfate compared with healthy older cats [33]. In the present study, plasma levels of 3-indoxyl sulfate were lower with the test food, consistent with a prior study of foods of a similar formulation to the one tested here [14]. Indoxyl sulfate, along with several other metabolites in this study, are among the uremic solutes that were found in patients on hemodialysis in at least 2.4-times higher levels compared with controls [34]. These included 3-hydroxyhippurate, 3-hydroxyphenylacetate sulfate, urea, dimethylarginine (SDMA + ADMA), 1-methylguanidine, 2-oxindole-3-acetate, 5-hydroxyindole sulfate, 6-hydroxyindole sulfate, and 4-ethylphenyl sulfate. In addition, 3-indoxyl sulfate, 5-hydroxyindole sulfate, 6-hydroxyindole sulfate, 3-hydroxyhippurate, 4-ethylphenyl sulfate, phenylpropionylglycine, and 3-phenylpropionate (hydrocinnamate) were all found at higher levels in cats with CKD compared with healthy controls [25]. As in the present study, lower levels of SDMA were also seen in the prior study of similarly formulated foods [14]. Serum SDMA can be used to identify CKD in cats, allowing earlier detection of CKD than serum creatinine [15], and its levels also increase with age in cats [28]. Both creatinine and BUN were significantly lower following consumption of the test food, and these were lower in healthy cats compared with those with CKD [25].

Several other metabolites that were at lower levels with the test food are also associated with kidney dysfunction, such as the urea cycle metabolites citrulline, urea, and dimethylarginine. Others include tryptophan and tyrosine metabolites, some of which can act as uremic toxins [35], and methylguanidine, which was seen at higher levels in plasma and urinary excretion in dogs with chronic renal failure [36]. Plasma levels of guanidinoacetate, a precursor to creatinine, were also lower following consumption of the test food in the present study. Erythronate, which was higher in people with CKD compared with those without CKD [37], was lower in plasma from cats fed the test food compared with the control food. Higher median concentrations of erythronate were also observed with older age [37].

Metabolites of tocopherols, which are vitamin E compounds with antioxidant activity [38], were higher in both serum and feces from cats fed the test food compared with the control food. A prior study showed that levels of vitamin E were lower in patients with CKD compared with healthy controls [39]. Similarly, significantly lower concentrations of gamma-tocopherol/beta-tocopherol were observed in cats with CKD compared with healthy cats [25].

Several of the OTUs that were of greater abundance with the test food in this study may have beneficial health effects concerning renal function. *Megasphaera* were at greater abundance in healthy controls than in people with CKD or idiopathic nephrotic syndrome [40]. Lactobacillaceae were significantly decreased in rats with chronic renal failure [41] and in people with end-stage renal disease [42] compared with healthy controls. *Dialister* was one of several genera that negatively correlated with CKD severity in humans, and also negatively correlated with the uremic toxins indoxyl sulfate and *p*-cresyl sulfate [43]. Consistent with those results, *Dialister* negatively correlated with 3-indoxyl sulfate in the present study. Similarly, *Bifidobacterium* was present at lower levels in feces from patients with ESRD compared with healthy controls and was negatively correlated with several renal parameters (cystatin C, BUN, creatinine, and estimated GFR) [44]. *Bifidobacterium* and Lactobacillaceae may confer beneficial effects on kidney function by upregulating IL-10, leading to decreased inflammation [32]. *Lactobacillus salivarius* prevented acute kidney injury in a cisplatin-induced rat model and lowered serum levels of the uremic toxins indoxyl sulfate and *p*-cresol sulfate [45]. It also improved intestinal permeability and led to an increase in fecal SCFAs. A mix of *L. paracasei* and *L. plantarum* showed reduced serum levels of the uremic toxins *p*-cresol, indoxyl sulfate, and *p*-cresyl sulfate in a CKD mouse model [46]. In addition, these probiotics appeared to improve intestinal barrier integrity, prevented kidney structural damage, decreased inflammation, and remediated CKD-related gut dysbiosis. A similar study on humans on hemodialysis who were given *L. rhamnosus* as a probiotic in a four-week clinical trial showed significantly decreased levels of the serum uremic toxins phenol and *p*-cresol compared with patients in the placebo group [47].

In contrast, a number of the OTUs that were of greater abundance with the control food appear to be associated with kidney dysfunction. *Paraprevotella* were at greater levels in the fecal microbiota of patients with CKD compared with healthy controls, and also correlated with estimated GFR, an indicator of CKD severity [48]. Similarly, Enterobacteriaceae was one of several families of greater abundance in the gut microbiota in people with kidney disease compared with healthy controls [42,49].

Advanced glycation end product (AGE) accumulation in body tissues is a characteristic of aging, as well as in diabetes and CKD [50]. In addition, oxidative stress is related to AGE accumulation, which is associated with age-related complications such as osteoarthritis and likely contributes to age-related loss of muscle mass [51]. Here, the AGE pyrraline was lower in serum and feces of cats fed the test food in this study compared with the control food.

Greater levels of dipeptides seen here in feces from cats fed the control food may indicate a shift toward proteolytic metabolism in the gut microbiota. In this study, true protein digestibility was 4% higher with the test food, so this, along with the presence of the fiber blend, could have contributed to the increased saccharolysis observed with the test food. Although the cats in the present study were all healthy, excess protein in CKD is delivered to the large intestine, which leads to a shift from saccharolytic to proteolytic bacteria. The resultant increased protein fermentation produces potentially detrimental metabolites such as indoles, phenols, ammonia, and amines [52]. As noted above, many detrimental uremic metabolites were lower with the test food, and were inversely correlated with OTUs that were of greater abundance with the test food. Cats with CKD showed higher levels of plasma indole sulfates and other uremic toxins with increased protein consumption [53]. Like the uremic metabolites, the same trend of lower levels with the test food and inverse correlation with OTUs of greater abundance with the test food was observed with dipeptides. Several bacterial families that were at greater levels in feces in cats fed the control food compared with the test food have been shown to generate phenolic compounds in vitro, including Clostridiaceae, Enterobacteriaceae, Lachnospiraceae, Porphyromonadaceae, and Veillonellaceae [54].

The SCFA butyric acid was higher and the BCFAs isobutryic acid and isovaleric acid were lower with the test food in this study. Lower levels of SCFAs and higher levels of BCFAs have been observed with greater levels of proteolysis and gut microbe-mediated putrefaction [24], further supporting the idea of a shift to proteolysis with the control food in the present study. SCFAs, including butyrate, provide several important functions such as serving as a major energy source for colonocytes [55]), protecting the integrity of the intestinal barrier [56,57], and inhibiting histone deacetylases, thus modulating oncologic and inflammatory functions [58]. While BCFAs have some beneficial properties as well, fecal isovaleric acid levels were significantly higher in cats with CKD compared with healthy controls, and fecal isovaleric acid was also found to correlate with creatinine levels [59].

In addition, a number of gamma-glutamyl amino acids were present at higher levels in plasma and feces from cats fed the control food. Their higher levels may indicate greater gamma-glutamyl transferase activity, which has been linked with increased oxidative stress and higher risk of cardiovascular disease [60].

Increases in metabolism of several amino acids, including tryptophan, tyrosine, valine, and lysine, have been associated with aging in humans [61]. Greater levels of metabolites of all of these pathways were observed in the present study in plasma and/or feces in cats fed the control food, contributing to the idea that the test food promoted anti-aging.

Some OTUs in this study have previously been associated with aging and/or cognition. *B. adolescentis* is of lower abundance in the gut microbiome of older adult humans [62,63], so it is of particular interest that feeding the test food in the present study appeared to increase the levels of this age-related species. *Coprococcus*, here at lower abundance with the test food, was present at significantly higher levels in feces from elderly (aged > 60 years) compared with middle-aged (aged 50–59 years) people [3], perhaps implying that the control food is associated with an increase in an age-associated microbe. Several *Lactobacillus* strains showed benefits in aging and age-induced metabolism by inhibition of telomere shortening and improving lipid, renal, and liver profiles in rats [64]. Porphyromonadaceae and Enterobacteriaceae, both of lower abundance with the test food, have been associated with cognitive decline in elderly humans [65]. The abundance of Enterobacteriaceae in the gut microbiome was higher in patients with post-stroke cognitive impairment and could distinguish between patients with and without cognitive impairment following a stroke [66]. In addition, aged mice showed higher anxiety-like behavior compared with younger mice, which directly correlated with the higher levels of Porphyromonadaceae in the ceca of older mice [67].

In the present study, levels of NAD^+^ were higher in feces from cats fed the test food, and nicotinate ribonucleoside, a precursor in the production of NAD^+^, was found at greater levels in plasma from cats fed the test food. NAD^+^ is an important coenzyme that carries out redox reactions in all cells and is particularly important for the generation of ATP. NAD^+^ also serves non-redox roles, including as a cofactor for poly (ADP-ribose) polymerase, which repairs oxidative damage to DNA. Decreased levels of NAD^+^, along with increased oxidative stress, have been seen in children with autism [68].

Bile salts also showed distinct patterns in this study, with the primary bile salt cholate at higher levels while several secondary bile salts were lower in feces from cats fed the test food. Notably, OTUs that were of greater abundance with the test food showed positive correlations with cholate and negative correlations with the secondary bile salts. *C. hiranonis*, of greater abundance with the control food, exhibits bile salt 7α-dehydroxylating activity [69], which also corresponds with the higher levels of deoxycholate and lithocholate seen in feces from cats fed the control food in this study.

Whether the levels of primary and secondary bile salts are beneficial or detrimental may be dependent upon situational differences. While secondary bile salts have been correlated with markers of colorectal cancer in humans [70], they also inhibit the growth of pathogens such as *Clostridioides difficile* [71,72]. Further, in dogs with chronic inflammatory enteropathy that experienced remission with nutritional therapy, higher levels of the secondary bile salts deoxycholate and lithocholate were observed along with elevated abundance of *C. hiranonis* during remission [73]. The authors of that study suggest that food therapy may have differential effects determined by the state of the gut microbiota.

Along this line of thought, not all of the OTUs of higher abundance following consumption of the control food are associated with detrimental functions. For example, *Adlercreutzia equolifaciens* can convert the isoflavone daidzein to equol [74], an isoflavan with beneficial health effects. Although the species of the *Adlercreutzia* OTU was not identified in the present study, equol was significantly higher in feces from cats fed the control food. In addition, Porphyromonadaceae and Enterobacteriaceae were positively correlated with albumin in a study of the gut microbiota of people with idiopathic nephrotic syndrome [75]. Porphyromonadaceae were also enriched in the gut microbiota of some elderly people and may modulate adiposity [76]. Further, although a number of metabolites and OTUs associated with poor health were higher with the control food, it is important to note that no adverse effects on body condition or general health were seen with either the control or test food in the senior cats in this study. However, it would be of interest to evaluate the test food in cats with a condition such as CKD to examine whether it would confer positive health effects.

Limitations of this study include the relatively short, 30-day feeding periods. While a number of changes in the plasma and fecal metabolites were observed, perhaps there would have been greater changes in the fecal microbiota with a longer feeding period. In addition, since the control and test foods differed in several ingredients, it is not possible to attribute the observed effects to a single ingredient. Rather, the conclusions must be drawn from the combined benefits of the ingredients (brown rice, oat groats, fiber blend with broccoli and tomato pomace) used together. Future studies may further investigate the individual effects of these ingredients.

## 5. Conclusions

This study showed that consumption of the test food resulted in higher levels of metabolites and microbiota associated with beneficial health states compared with the control food. Metabolism with the test food appeared to favor saccharolysis in contrast with proteolysis favored with the control food. Positive correlations with metabolites involved in saccharolysis and negative correlations with uremic toxins, amino acid metabolism, and BCFAs support the idea that the test food may support the health of senior cats.

## Figures and Tables

**Table 1:** Proximate analysis of foods used in this study.

Analyte (%)	Control Food	Test Food
Ash	4.66	5.18
Crude fat	19.08	16.80
Crude fiber	1.40	1.30
Crude protein	32.71	31.18
Moisture	6.43	5.17
Omega-3, sum	1.05	0.44
Omega-6, sum	3.64	3.82

**Table 2:** Digestibility analyses.

	Control Food	Test Food
Apparent dry matter digestibility	84.1	85.6
Apparent protein digestibility	85.2	88.5
True protein digestibility	91.8	95.8
Apparent fat digestibility	93.1	92.2
Apparent fiber digestibility	31.2	15.5
Apparent carbohydrate digestibility	88.0	88.0
Apparent vital nutrient digestibility	90.4	91.4
Apparent energy digestibility	86.6	87.9
Gross energy, kcal/kg	5129	5088
Digestible energy, kcal/kg	4442	4471
Metabolizable energy, kcal/kg	4207	4236
Neutral detergent fiber calories	31.8	35.4
Protein calories	29.6	29.1
Fat calories	38.6	35.5

Data are presented as mean percentage unless otherwise indicated.

**Table 3:** Body weight, intake, and stool score.

				Mean Difference		
	Baseline	Control Food	Test Food	Control Food−Baseline	Test Food−Baseline	Test Food−Control Food
Body weight, kg	4.40 ± 0.12	4.57 ± 0.13	4.59 ± 0.13	0.13 ± 0.04 *p* = 0.001	0.16 ± 0.03 *p* < 0.001	0.02 ± 0.04 *p* = 0.600
Average daily intake, g	49.3 ± 2.17	51.5 ± 1.98	59.4 ± 2.6	2.18 ± 0.9 *p* = 0.02	10.1 ± 1.1 *p* = 0.001	7.9 ± 1.2 *p* < 0.001
Average stool score	4.9 ± 0.04	4.9 ± 0.04	4.6 ± 0.12	0 ± 0.06 *p* = 0.500	−0.33 ± 0.11 *p* = 0.006	−0.33 ± 0.13 *p* = 0.010

Data are presented as mean ± standard error for all 40 cats. Stool was scored on a 1–5 scale.

**Table 4:** Blood chemistry parameters.

	Control Food	Test Food	*p* Value
Creatinine, mg/dL	1.22	1.15	<0.001
BUN, mg/dL	25.44	23.92	<0.001
Albumin, g/dL	2.98	3.05	0.060
Total protein, g/dL	7.18	7.31	0.190

BUN, blood urea nitrogen.

**Table 5:** Plasma metabolites of interest following consumption of study foods by senior cats.

Plasma Metabolite	Control Food–Test Food	*p* Value
Amino acid metabolism		
Creatinine metabolism		
guanidinoacetate	0.43 ± 0.07	<0.001
Dipeptide derivative		
N-acetylcarnosine	0.25 ± 0.07	0.001
Gamma-glutamyl amino acid		
gamma-glutamylglutamine	0.11 ± 0.02	<0.001
gamma-glutamylglycine	0.22 ± 0.03	<0.001
gamma-glutamylleucine	0.17 ± 0.03	<0.001
gamma-glutamylmethionine	0.19 ± 0.04	<0.001
gamma-glutamylvaline	0.12 ± 0.03	<0.001
Guanidino and acetamido metabolism		
1-methylguanidine	0.25 ± 0.07	0.001
Leucine, isoleucine, and valine metabolism		
alpha-hydroxyisovalerate	0.10 ± 0.03	<0.001
Methionine, cysteine, SAM, taurine metabolism		
S-methylcysteine sulfoxide	0.17 ± 0.2	<0.001
Tryptophan metabolism		
3-indoxyl sulfate	0.84 ± 0.17	<0.001
5-hydroxyindole sulfate	0.82 ± 0.21	<0.001
6-hydroxyindole sulfate	0.92 ± 0.19	<0.001
Tyrosine metabolism		
dopamine-3-O-sulfate	0.19 ± 0.06	0.001
3-hydroxyphenylacetate sulfate	0.48 ± 0.14	0.001
Urea cycle; arginine and proline metabolism		
citrulline	0.13 ± 0.04	0.001
dimethylarginine (sdma + adma)	0.07 ± 0.02	<0.001
urea	0.08 ± 0.02	0.001
Carbohydrate metabolism		
Aminosugar metabolism		
erythronate	0.11 ± 0.03	<0.001
Glycolysis, gluconeogenesis, and pyruvate metabolism		
1,5-anhydroglucitol	0.24 ± 0.06	0.001
glycerate	0.12 ± 0.03	<0.001
Pentose metabolism		
arabitol/xylitol	0.21 ± 0.05	<0.001
Cofactors and vitamins		
Nicotinate and nicotinamide metabolism		
nicotinate ribonucleoside	−1.27 ± 0.12	<0.001
trigonelline (N’-methylnicotinate)	−1.95 ± 0.02	<0.001
Tocopherol metabolism		
alpha-CEHC sulfate	−0.44 ± 0.08	<0.001
gamma-tocopherol/beta-tocopherol	−0.66 ± 0.05	<0.001
Lipid metabolism		
Secondary bile salt metabolism		
taurolithocholate-3-sulfate	0.50 ± 0.08	<0.001
ursocholate	−1.03 ± 0.22	<0.001
Xenobiotics		
Benzoate metabolism		
3-hydroxyhippurate	0.58 ± 0.16	0.001
3-phenylpropionate (hydrocinnamate)	0.97 ± 0.21	<0.001
4-ethylphenyl sulfate	1.51 ± 0.18	<0.001
phenylpropionylglycine	1.10 ± 0.24	<0.001
Food component/plant		
2-oxindole-3-acetate	0.58 ± 0.17	0.001
indolin-2-one	0.70 ± 0.17	<0.001
pyrraline	0.32 ± 0.09	0.001

Values are the mean of the difference of the control and test foods ± standard error. ADMA, asymmetric dimethylarginine; CEHC, 2-(β-carboxyethyl)-6-hydroxychroman; SAM, S-adenosyl methionine; SDMA, symmetric dimethylarginine.

**Table 6:** Fecal metabolites of interest following consumption of study foods by senior cats.

Fecal Metabolite	Control Food–Test Food	*p* Value
Amino acid metabolism		
Alanine and aspartate metabolism		
N-methylalanine	0.73 ± 0.12	<0.001
N-propionylalanine	0.76 ± 0.10	<0.001
propionylglutamine	0.92 ± 0.13	<0.001
Dipeptide		
glycylisoleucine	0.79 ± 0.13	<0.001
glycylleucine	0.51 ± 0.10	<0.001
glycylvaline	0.65 ± 0.11	<0.001
isoleucylglycine	0.97 ± 0.18	<0.001
leucylglycine	1.00 ± 0.20	<0.001
threonylphenylalanine	0.95 ± 0.20	<0.001
valylleucine	1.04 ± 0.20	<0.001
Gamma-glutamyl amino acid		
gamma-glutamylleucine	0.78 ± 0.15	<0.001
Glutamate metabolism		
carboxyethyl-GABA	0.89 ± 0.03	<0.001
propionylglycine	0.78 ± 0.17	<0.001
Histidine metabolism		
cis-urocanate	0.85 ± 0.16	<0.001
trans-urocanate	0.95 ± 0.18	<0.001
Leucine, isoleucine, and valine metabolism		
3-methylglutaconate	0.36 ± 0.07	< 0.001
Lysine metabolism		
N,N,N-trimethyl-5-aminovalerate	0.40 ± 0.07	<0.001
Polyamine metabolism		
N-acetyl-isoputreanine	0.61 ± 0.05	<0.001
spermidine	1.20 ± 0.15	<0.001
Tryptophan metabolism		
indolelactate	−0.91 ± 0.17	<0.001
serotonin	0.58 ± 0.09	<0.001
tryptamine	0.74 ± 0.12	<0.001
tryptophan betaine	0.68 ± 0.13	<0.001
Carbohydrate metabolism		
Aminosugar metabolism		
erythronate	−0.79 ± 0.15	<0.001
glucuronate	−0.62 ± 0.12	<0.001
N-acetylglucosaminylasparagine	−1.27 ± 0.24	<0.001
Disaccharides and oligosaccharides		
Lactose	−1.35 ± 0.26	<0.001
Fructose, mannose, and galactose metabolism		
galactonate	−1.47 ± 0.23	<0.001
mannitol/sorbitol	−1.90 ± 0.34	<0.001
Glycogen metabolism		
maltol	−2.10 ± 0.15	<0.001
maltose	−1.05 ± 0.19	<0.001
maltotetraose	−1.71 ± 0.28	<0.001
maltotriose	−1.14 ± 0.20	<0.001
Glycolysis, gluconeogenesis, and pyruvate metabolism		
glucose	−0.71 ± 0.10	<0.001
lactate	−1.24 ± 0.25	<0.001
Pentose metabolism		
arabitol/xylitol	−1.21 ± 0.25	<0.001
arabinose	−1.20 ± 0.15	<0.001
arabonate/xylonate	−1.11 ± 0.24	<0.001
ribonate (ribonolactone)	−1.11 ± 0.19	<0.001
ribulose/xylulose	−1.10 ± 0.17	<0.001
sedoheptulose	−0.43 ± 0.09	<0.001
xylose	2.18 ± 0.13	<0.001
Cofactors and vitamins		
Ascorbate and aldarate metabolism		
threonate	−1.26 ± 0.17	<0.001
Nicotinate and nicotinamide metabolism		
NAD^+^	−1.27 ± 0.23	<0.001
nicotinate	0.32 ± 0.07	0.020
NaMN	−0.57 ± 0.12	<0.001
trigonelline (N’-methylnicotinate)	−0.90 ± 0.10	<0.001
Riboflavin metabolism		
flavin adenine dinucleotide (FAD)	−0.57 ± 0.11	<0.001
riboflavin (vitamin B_2_)	−1.03 ± 0.09	<0.001
Thiamine metabolism		
hydroxymethylpyrimidine	−0.65 ± 0.10	<0.001
thiamin (vitamin b_1_)	−0.44 ± 0.06	<0.001
thiamin monophosphate	−0.96 ± 0.13	<0.001
Tocopherol metabolism		
alpha-CEHC sulfate	−1.68 ± 0.31	<0.001
alpha-tocotrienol	−0.34 ± 0.05	<0.001
delta-tocopherol	−0.22 ± 0.03	<0.001
gamma-CEHC sulfate	−1.87 ± 0.24	<0.001
gamma-tocopherol/beta-tocopherol	−0.69 ± 0.03	<0.001
Vitamin B_6_ metabolism		
pyridoxate	−0.51 ± 0.04	<0.001
pyridoxine (vitamin b_6_)	−1.12 ± 0.16	<0.001
Lipid metabolism		
Primary bile salt metabolism		
cholate	−0.77 ± 0.16	<0.001
Secondary bile salt metabolism		
7-alpha-hydroxycholestenone	1.05 ± 0.06	<0.001
dehydrolithocholate	1.95 ± 0.23	<0.001
deoxycholate	1.06 ± 0.14	<0.001
isoursodeoxycholate	0.83 ± 0.10	<0.001
lithocholate	1.76 ± 0.17	<0.001
ursodeoxycholate	0.78 ± 0.13	<0.001
Xenobiotics		
Food component/plant		
2-oxindole-3-acetate	0.38 ± 0.06	<0.001
indolin-2-one	1.70 ± 0.24	<0.001
pyrraline	0.47 ± 0.09	<0.001

Values are the mean of the difference of the control and test foods ± standard error. CEHC, 2-(β-carboxyethyl)-6-hydroxychroman; FAD, flavin adenine dinucleotide; GABA, gamma aminobutyric acid; NAD, nicotinamide adenine dinucleotide; NaMN, nicotinic acid mononucleotide.

**Table 7:** Fecal short-chain fatty acids following consumption of the study foods.

	Control Food	Test Food	*p* Value
Short-chain fatty acids, ppm			
Acetic acid	3646.7	3889.5	0.410
Butyric acid	2641.8	4807.8	<0.001
Propionic acid	1529.7	850.7	<0.001
Branched-chain fatty acids, ppm			
Isobutryic acid	214.6	147.5	<0.001
Isovaleric acid	300.9	212.2	<0.001

ppm, parts per million.

**Table 8:** Correlations between plasma indoles and OTUs.

	Estimate ± SE	*p* Value	r^2^
2-oxindole-3-acetate			
1084643 Mogibacteriaceae *Mogibacterium*	0.93 ± 0.29	0.002	0.14
839684 Lachnospiraceae unclassified	0.90 ± 0.27	0.001	0.15
100212 Veillonellaceae	0.79 ± 0.26	0.003	0.13
4384058 Enterobacteriaceae *Salmonella*	0.75 ± 0.25	0.004	0.12
193279 Coriobacteriaceae *Adlercreutzia*	0.72 ± 0.25	0.006	0.11
266392 Lachnospiraceae *Coprococcus*	0.68 ± 0.25	0.008	0.10
100027 Enterobacteriaceae	0.47 ± 0.19	0.019	0.08
356403 Clostridiaceae *Clostridium hiranonis*	0.44 ± 0.18	0.020	0.08
100001 unclassified	−0.26 ± 0.11	0.026	0.07
3-indoxyl sulfate			
193279 Coriobacteriaceae *Adlercreutzia*	0.94 ± 0.18	<0.001	0.29
266392 Lachnospiraceae *Coprococcus*	0.87 ± 0.18	<0.001	0.26
4384058 Enterobacteriaceae *Salmonella*	0.66 ± 0.20	0.002	0.14
1084643 Mogibacteriaceae *Mogibacterium*	0.52 ± 0.24	0.036	0.07
1000148 Comamonadaceae unclassified	0.46 ± 0.15	0.004	0.12
356403 Clostridiaceae *Clostridium hiranonis*	0.44 ± 0.14	0.004	0.12
4319416 Bartonellaceae *Bartonella* unclassified	−0.25 ± 0.12	0.035	0.07
100001 unclassified	−0.25 ± 0.09	0.007	0.11
128382 Veillonellaceae *Dialister*	−0.46 ± 0.15	0.004	0.12
4441081 Coriobacteriaceae unclassified	−0.47 ± 0.14	0.001	0.16
52166 Veillonellaceae *Megasphaera*	−0.53 ± 0.16	0.001	0.15
652696 Bifidobacteriaceae *Bifidobacterium adolescentis*	−0.58 ± 0.23	0.014	0.09
5-hydroxyindole sulfate			
193279 Coriobacteriaceae *Adlercreutzia*	0.71 ± 0.16	<0.001	0.24
266392 Lachnospiraceae *Coprococcus*	0.68 ± 0.15	<0.001	0.24
4384058 Enterobacteriaceae *Salmonella*	0.51 ± 0.17	0.003	0.12
1000148 Comamonadaceae unclassified	0.36 ± 0.13	0.005	0.11
356403 Clostridiaceae Clostridium *hiranonis*	0.29 ± 0.12	0.021	0.08
100001 unclassified	−0.19 ± 0.07	0.011	0.10
4319416 Bartonellaceae Bartonella unclassified	−0.24 ± 0.09	0.014	0.09
4441081 Coriobacteriaceae unclassified	−0.40 ± 0.11	0.001	0.16
128382 Veillonellaceae *Dialister*	−0.41 ± 0.12	0.001	0.15
52166 Veillonellaceae *Megasphaera*	−0.43 ± 0.13	0.002	0.14
652696 Bifidobacteriaceae *Bifidobacterium adolescentis*	−0.50 ± 0.19	0.010	0.10
6-hydroxyindole sulfate			
193279 Coriobacteriaceae *Adlercreutzia*	0.77 ± 0.16	<0.001	0.28
266392 Lachnospiraceae *Coprococcus*	0.76 ± 0.15	<0.001	0.28
4384058 Enterobacteriaceae *Salmonella*	0.56 ± 0.17	0.002	0.14
1084643 Mogibacteriaceae *Mogibacterium*	0.46 ± 0.20	0.027	0.07
1000148 Comamonadaceae unclassified	0.41 ± 0.13	0.002	0.14
356403 Clostridiaceae *Clostridium hiranonis*	0.34 ± 0.12	0.006	0.11
100001 unclassified	−0.20 ± 0.08	0.011	0.10
4319416 Bartonellaceae *Bartonella* unclassified	−0.23 ± 0.10	0.021	0.08
4441081 Coriobacteriaceae unclassified	−0.41 ± 0.11	0.001	0.16
128382 Veillonellaceae *Dialister*	−0.42 ± 0.13	0.002	0.14
52166 Veillonellaceae *Megasphaera*	−0.43 ± 0.13	0.002	0.14
652696 Bifidobacteriaceae *Bifidobacterium adolescentis*	−0.51 ± 0.19	0.009	0.10
Indolin-2-one			
193279 Coriobacteriaceae *Adlercreutzia*	0.84 ± 0.23	<0.001	0.17
266392 Lachnospiraceae *Coprococcus*	0.80 ± 0.23	0.001	0.16
4384058 Enterobacteriaceae *Salmonella*	0.68 ± 0.24	0.006	0.11
356403 Clostridiaceae *Clostridium hiranonis*	0.51 ± 0.17	0.004	0.12
1000148 Comamonadaceae unclassified	0.39 ± 0.18	0.034	0.07
100001 unclassified	−0.25 ± 0.10	0.019	0.08
4441081 Coriobacteriaceae unclassified	−0.53 ± 0.16	0.001	0.15
128382 Veillonellaceae *Dialister*	−0.55 ± 0.18	0.003	0.13
652696 Bifidobacteriaceae *Bifidobacterium adolescentis*	−0.56 ± 0.27	0.042	0.06

Operational taxonomic unit (OTU) number, family, and genus are shown; species are also shown when indicated. r^2^, square of Pearson’s correlation coefficient; SE, standard error.

**Table 9:** Correlations between fecal metabolites and OTUs.

	Estimate ± SE		*p* Value	r^2^
Indoles				
2-oxindole-3-acetate				
839684 Lachnospiraceae unclassified	2.38 ± 0.68		0.001	0.16
356403 Clostridiaceae *Clostridium hiranonis*	2.37 ± 0.38		<0.001	0.37
4384058 Enterobacteriaceae *Salmonella*	2.34 ± 0.62		<0.001	0.18
1084643 Mogibacteriaceae *Mogibacterium*	2.32 ± 0.74		0.003	0.13
100027 Enterobacteriaceae	1.85 ± 0.46		<0.001	0.20
266392 Lachnospiraceae *Coprococcus*	1.70 ± 0.63		0.009	0.10
1000148 Comamonadaceae unclassified	1.43 ± 0.48		0.004	0.12
1758401 Clostridiaceae *SMB53*	1.30 ± 0.37		0.001	0.16
100001 unclassified	−0.87 ± 0.28		0.002	0.14
4441081 Coriobacteriaceae unclassified	−1.22 ± 0.44		0.007	0.11
52166 Veillonellaceae *Megasphaera*	−1.41 ± 0.52		0.009	0.10
128382 Veillonellaceae *Dialister*	−1.41 ± 0.48		0.005	0.12
100100 Bacillaceae unclassified	−1.43 ± 0.49		0.004	0.12
Indolelactate				
128382 Veillonellaceae *Dialister*	0.55 ± 0.16		0.001	0.16
1007180 Methylobacteriaceae *Methylobacterium*	0.22 ± 0.07		0.003	0.13
1074945 Propionibacteriaceae *Propionibacterium granulosum*	0.22 ± 0.07		0.003	0.13
2170756 Xanthomonadaceae *Stenotrophomonas geniculata*	0.22 ± 0.07		0.003	0.13
579304 Lactobacillaceae	0.22 ± 0.07		0.003	0.13
356403 Clostridiaceae *Clostridium hiranonis*	−0.41 ± 0.15		0.010	0.10
1105591 Paraprevotellaceae *Paraprevotella*	−0.45 ± 0.13		0.001	0.15
1000148 Comamonadaceae unclassified	−0.51 ± 0.16		0.002	0.14
266392 Lachnospiraceae *Coprococcus*	−0.60 ± 0.21		0.006	0.11
4384058 Enterobacteriaceae *Salmonella*	−0.64 ± 0.21		0.004	0.12
839684 Lachnospiraceae unclassified	−0.69 ± 0.23		0.004	0.12
193279 Coriobacteriaceae *Adlercreutzia*	−0.84 ± 0.20		<0.001	0.21
1084643 Mogibacteriaceae *Mogibacterium*	−0.90 ± 0.24		<0.001	0.18
Indolin-2-one				
1084643 Mogibacteriaceae *Mogibacterium*	1.00 ± 0.16		<0.001	0.39
193279 Coriobacteriaceae *Adlercreutzia*	0.99 ± 0.12		<0.001	0.52
4384058 Enterobacteriaceae *Salmonella*	0.73 ± 0.15		<0.001	0.28
1000148 Comamonadaceae unclassified	0.72 ± 0.09		<0.001	0.48
266392 Lachnospiraceae *Coprococcus*	0.70 ± 0.14		<0.001	0.27
356403 Clostridiaceae *Clostridium hiranonis*	0.52 ± 0.10		<0.001	0.28
839684 Lachnospiraceae unclassified	0.47 ± 0.18		0.010	0.10
1105591 Paraprevotellaceae *Paraprevotella*	0.32 ± 0.10		0.002	0.14
1000062 Porphyromonadaceae unclassified	0.29 ± 0.10		0.004	0.12
4473250 Paenibacillaceae *Aneurinibacillus migulanus*	−0.18 ± 0.06		0.004	0.12
1007180 Methylobacteriaceae *Methylobacterium*	−0.21 ± 0.05		<0.001	0.22
1074945 Propionibacteriaceae *Propionibacterium granulosum*	−0.21 ± 0.05		<0.001	0.22
2170756 Xanthomonadaceae *Stenotrophomonas geniculata*	−0.21 ± 0.05		<0.001	0.22
579304 Lactobacillaceae	−0.21 ± 0.05		<0.001	0.22
100001 unclassified	−0.32 ± 0.06		<0.001	0.28
4319416 Bartonellaceae *Bartonella* unclassified	−0.40 ± 0.08		<0.001	0.28
52166 Veillonellaceae *Megasphaera*	−0.43 ± 0.13		0.001	0.15
4441081 Coriobacteriaceae unclassified	−0.52 ± 0.10		<0.001	0.30
652696 Bifidobacteriaceae *Bifidobacterium adolescentis*	−0.57 ± 0.18		0.002	0.14
128382 Veillonellaceae *Dialister*	−0.63 ± 0.10		<0.001	0.37
Bile salts				
Cholate				
128382 Veillonellaceae *Dialister*	0.78 ± 0.18		<0.001	0.22
4441081 Coriobacteriaceae unclassified	0.66 ± 0.17		<0.001	0.20
4319416 Bartonellaceae *Bartonella* unclassified	0.49 ± 0.14		0.001	0.17
100001 unclassified	0.41 ± 0.11		<0.001	0.18
1007180 Methylobacteriaceae *Methylobacterium*	0.37 ± 0.08		<0.001	0.27
1074945 Propionibacteriaceae *Propionibacterium granulosum*	0.37 ± 0.08		<0.001	0.27
2170756 Xanthomonadaceae *Stenotrophomonas geniculata*	0.37 ± 0.08		<0.001	0.27
579304 Lactobacillaceae	0.37 ± 0.08		<0.001	0.27
356403 Clostridiaceae *Clostridium hiranonis*	−0.61 ± 0.18		0.001	0.15
1000148 Comamonadaceae unclassified	−0.75 ± 0.18		<0.001	0.21
4384058 Enterobacteriaceae *Salmonella*	−0.77 ± 0.26		0.004	0.12
266392 Lachnospiraceae *Coprococcus*	−0.91 ± 0.24		<0.001	0.18
1084643 Mogibacteriaceae *Mogibacterium*	−1.20 ± 0.28		<0.001	0.22
193279 Coriobacteriaceae *Adlercreutzia*	−1.24 ± 0.23		<0.001	0.32
Dehydrolithocholate				
193279 Coriobacteriaceae *Adlercreutzia*	0.82 ± 0.13		<0.001	0.39
1084643 Mogibacteriaceae *Mogibacterium*	0.67 ± 0.17		<0.001	0.19
4384058 Enterobacteriaceae *Salmonella*	0.66 ± 0.14		<0.001	0.26
266392 Lachnospiraceae *Coprococcus*	0.65 ± 0.14		<0.001	0.27
356403 Clostridiaceae *Clostridium hiranonis*	0.62 ± 0.09		<0.001	0.45
839684 Lachnospiraceae unclassified	0.61 ± 0.16		<0.001	0.19
1000148 Comamonadaceae unclassified	0.57 ± 0.10		<0.001	0.34
100212 Veillonellaceae	0.52 ± 0.15		0.001	0.15
100027 Enterobacteriaceae	0.46 ± 0.11		<0.001	0.22
1758401 Clostridiaceae *SMB53*	0.25 ± 0.09		0.009	0.10
4473250 Paenibacillaceae *Aneurinibacillus migulanus*	−0.21 ± 0.05		<0.001	0.18
4319416 Bartonellaceae *Bartonella* unclassified	−0.24 ± 0.08		0.007	0.11
100001 unclassified	−0.28 ± 0.06		<0.001	0.25
52166 Veillonellaceae *Megasphaera*	−0.39 ± 0.12		0.002	0.14
100100 Bacillaceae unclassified	−0.39 ± 0.11		0.001	0.16
128382 Veillonellaceae *Dialister*	−0.44 ± 0.11		<0.001	0.20
Deoxycholate				
193279 Coriobacteriaceae *Adlercreutzia*	1.25 ± 0.27		<0.001	0.25
4384058 Enterobacteriaceae *Salmonella*	1.22 ± 0.27		<0.001	0.24
839684 Lachnospiraceae unclassified	1.00 ± 0.31		0.002	0.14
1000148 Comamonadaceae unclassified	0.97 ± 0.20		<0.001	0.27
100027 Enterobacteriaceae	0.94 ± 0.21		<0.001	0.25
356403 Clostridiaceae *Clostridium hiranonis*	0.88 ± 0.19		<0.001	0.25
266392 Lachnospiraceae *Coprococcus*	0.86 ± 0.28		0.003	0.13
1105591 Paraprevotellaceae *Paraprevotella*	0.54 ± 0.18		0.005	0.12
4473250 Paenibacillaceae *Aneurinibacillus migulanus*	−0.32 ± 0.11		0.005	0.12
100001 unclassified	−0.42 ± 0.12		0.001	0.15
4319416 Bartonellaceae *Bartonella* unclassified	−0.59 ± 0.15		<0.001	0.18
100100 Bacillaceae unclassified	−0.66 ± 0.22		0.004	0.12
128382 Veillonellaceae *Dialister*	−0.86 ± 0.21		<0.001	0.21
Isoursodeoxycholate				
839684 Lachnospiraceae unclassified	2.08 ± 0.44		<0.001	0.26
4384058 Enterobacteriaceae *Salmonella*	1.76 ± 0.41		<0.001	0.22
100212 Veillonellaceae	1.69 ± 0.43		<0.001	0.19
193279 Coriobacteriaceae *Adlercreutzia*	1.64 ± 0.42		<0.001	0.19
100027 Enterobacteriaceae	1.55 ± 0.30		<0.001	0.30
356403 Clostridiaceae *Clostridium hiranonis*	1.45 ± 0.28		<0.001	0.30
266392 Lachnospiraceae *Coprococcus*	1.23 ± 0.43		0.006	0.11
1000148 Comamonadaceae unclassified	1.06 ± 0.33		0.002	0.14
1105591 Paraprevotellaceae *Paraprevotella*	0.93 ± 0.27		0.001	0.16
1758401 Clostridiaceae *SMB53*	0.80 ± 0.26		0.003	0.13
4473250 Paenibacillaceae *Aneurinibacillus migulanus*	−0.49 ± 0.16		0.004	0.12
100001 unclassified	−0.66 ± 0.19		0.001	0.17
100100 Bacillaceae unclassified	−1.12 ± 0.33		0.001	0.16
128382 Veillonellaceae *Dialister*	−1.13 ± 0.32		0.001	0.16
Lithocholate				
193279 Coriobacteriaceae *Adlercreutzia*	0.98 ± 0.16		<0.001	0.36
1084643 Mogibacteriaceae *Mogibacterium*	0.83 ± 0.21		<0.001	0.20
4384058 Enterobacteriaceae *Salmonella*	0.83 ± 0.17		<0.001	0.26
266392 Lachnospiraceae *Coprococcus*	0.74 ± 0.17		<0.001	0.22
1000148 Comamonadaceae unclassified	0.70 ± 0.12		<0.001	0.33
839684 Lachnospiraceae unclassified	0.65 ± 0.20		0.002	0.14
356403 Clostridiaceae *Clostridium hiranonis*	0.60 ± 0.12		<0.001	0.27
100027 Enterobacteriaceae	0.50 ± 0.14		0.001	0.16
1105591 Paraprevotellaceae *Paraprevotella*	0.35 ± 0.12		0.004	0.12
1000062 Porphyromonadaceae unclassified	0.32 ± 0.12		0.007	0.11
1007180 Methylobacteriaceae *Methylobacterium*	−0.20 ± 0.06		0.002	0.14
1074945 Propionibacteriaceae *Propionibacterium granulosum*	−0.20 ± 0.06		0.002	0.14
2170756 Xanthomonadaceae *Stenotrophomonas geniculata*	−0.20 ± 0.06		0.002	0.14
579304 Lactobacillaceae	−0.20 ± 0.06		0.002	0.14
4473250 Paenibacillaceae *Aneurinibacillus migulanus*	−0.21 ± 0.07		0.003	0.13
100001 unclassified	−0.32 ± 0.08		<0.001	0.21
4319416 Bartonellaceae *Bartonella* unclassified	−0.41 ± 0.10		<0.001	0.21
52166 Veillonellaceae *Megasphaera*	−0.44 ± 0.15		0.006	0.11
4441081 Coriobacteriaceae unclassified	−0.44 ± 0.13		0.001	0.16
128382 Veillonellaceae *Dialister*	−0.66 ± 0.13		<0.001	0.29
Ursodeoxycholate				
839684 Lachnospiraceae unclassified	1.58 ± 0.43		<0.001	0.17
100212 Veillonellaceae	1.45 ± 0.41		0.001	0.16
4384058 Enterobacteriaceae *Salmonella*	1.43 ± 0.40		0.001	0.17
100027 Enterobacteriaceae	1.32 ± 0.29		<0.001	0.25
356403 Clostridiaceae *Clostridium hiranonis*	0.98 ± 0.28		0.001	0.16

Operational taxonomic unit (OUT) number, family, and genus are shown; species are also shown when indicated. r^2^, square of Pearson’s correlation coefficient; SE, standard error.

**Table 10:** Correlations between fecal short-chain fatty acids and OTUs.

	Estimate ± SE	*p* Value	r^2^
Short-chain fatty acid			
Acetic acid			
100001 unclassified	377.86 ± 169.92	0.029	0.06
193279 Coriobacteriaceae *Adlercreutzia*	−162.58 ± 69.07	0.021	0.07
266392 Lachnospiraceae *Coprococcus*	−180.36 ± 74.28	0.018	0.08
4384058 Enterobacteriaceae *Salmonella*	−186.24 ± 73.79	0.014	0.08
100027 Enterobacteriaceae	−257.88 ± 99.59	0.012	0.09
Butyric acid			
100001 unclassified	1481.79 ± 228.27	<0.001	0.37
1007180 Methylobacteriaceae *Methylobacterium*	1205.37 ± 327.19	<0.001	0.16
1074945 Propionibacteriaceae *Propionibacterium granulosum*	1205.37 ± 327.19	<0.001	0.16
2170756 Xanthomonadaceae *Stenotrophomonas geniculata*	1205.37 ± 327.19	<0.001	0.16
579304 Lactobacillaceae	1205.37 ± 327.19	<0.001	0.16
4473250 Paenibacillaceae *Aneurinibacillus migulanus*	1094.04 ± 311.20	0.001	0.15
52166 Veillonellaceae *Megasphaera*	834.55 ± 120.91	<0.001	0.40
4441081 Coriobacteriaceae unclassified	741.05 ± 153.17	<0.001	0.25
4319416 Bartonellaceae *Bartonella* unclassified	711.14 ± 188.01	<0.001	0.17
128382 Veillonellaceae *Dialister*	681.55 ± 139.49	<0.001	0.25
100100 Bacillaceae unclassified	506.90 ± 156.59	0.002	0.13
652696 Bifidobacteriaceae *Bifidobacterium adolescentis*	310.52 ± 115.20	0.009	0.09
1084643 Mogibacteriaceae *Mogibacterium*	−351.85 ± 94.69	<0.001	0.16
100212 Veillonellaceae	−361.32 ± 119.61	0.003	0.11
839684 Lachnospiraceae unclassified	−427.17 ± 112.65	<0.001	0.17
1000062 Porphyromonadaceae unclassified	−479.32 ± 185.41	0.012	0.08
1105591 Paraprevotellaceae *Paraprevotella*	−522.93 ± 173.04	0.003	0.11
266392 Lachnospiraceae *Coprococcus*	−529.60 ± 109.93	<0.001	0.24
4384058 Enterobacteriaceae *Salmonella*	−540.56 ± 108.67	<0.001	0.26
1758401 Clostridiaceae *SMB53*	−651.43 ± 174.01	<0.001	0.16
1000148 Comamonadaceae unclassified	−655.39 ± 137.87	<0.001	0.24
193279 Coriobacteriaceae *Adlercreutzia*	−667.17 ± 87.02	<0.001	0.45
100027 Enterobacteriaceae	−760.96 ± 144.85	<0.001	0.28
356403 Clostridiaceae *Clostridium hiranonis*	−876.23 ± 132.62	<0.001	0.38
Propionic acid			
356403 Clostridiaceae *Clostridium hiranonis*	281.28 ± 57.68	<0.001	0.25
1758401 Clostridiaceae *SMB53*	225.48 ± 70.43	0.002	0.12
1000148 Comamonadaceae unclassified	215.68 ± 57.15	<0.001	0.17
1000062 Porphyromonadaceae unclassified	206.39 ± 72.75	0.006	0.10
1105591 Paraprevotellaceae *Paraprevotella*	186.05 ± 69.32	0.009	0.09
193279 Coriobacteriaceae *Adlercreutzia*	169.61 ± 41.89	<0.001	0.19
100027 Enterobacteriaceae	154.64 ± 64.92	0.020	0.07
4384058 Enterobacteriaceae *Salmonella*	140.78 ± 47.01	0.004	0.11
839684 Lachnospiraceae unclassified	115.29 ± 46.91	0.016	0.08
266392 Lachnospiraceae *Coprococcus*	108.81 ± 48.36	0.028	0.07
1084643 Mogibacteriaceae *Mogibacterium*	81.80 ± 39.76	0.043	0.06
100100 Bacillaceae unclassified	−159.61 ± 63.61	0.014	0.08
652696 Bifidobacteriaceae *Bifidobacterium adolescentis*	−185.12 ± 42.57	<0.001	0.21
52166 Veillonellaceae *Megasphaera*	−202.27 ± 56.90	0.001	0.15
128382 Veillonellaceae *Dialister*	−249.93 ± 56.49	<0.001	0.21
4441081 Coriobacteriaceae unclassified	−269.82 ± 62.12	<0.001	0.21
4319416 Bartonellaceae *Bartonella* unclassified	−315.69 ± 72.48	<0.001	0.21
100001 unclassified	−322.04 ± 107.23	0.004	0.11
1007180 Methylobacteriaceae *Methylobacterium*	−338.04 ± 135.44	0.015	0.08
1074945 Propionibacteriaceae *Propionibacterium granulosum*	−338.04 ± 135.44	0.015	0.08
2170756 Xanthomonadaceae *Stenotrophomonas geniculata*	−338.04 ± 135.44	0.015	0.08
579304 Lactobacillaceae	−338.04 ± 135.44	0.015	0.08
Branched-chain fatty acid			
Isobutyric acid			
1105591 Paraprevotellaceae *Paraprevotella*	24.15 ± 6.64	0.001	0.16
1000148 Comamonadaceae unclassified	17.85 ± 5.84	0.003	0.12
1758401 Clostridiaceae *SMB53*	15.56 ± 7.32	0.037	0.06
839684 Lachnospiraceae unclassified	14.18 ± 4.52	0.003	0.12
100027 Enterobacteriaceae	14.14 ± 6.45	0.032	0.06
193279 Coriobacteriaceae *Adlercreutzia*	13.88 ± 4.27	0.002	0.13
4384058 Enterobacteriaceae *Salmonella*	10.98 ± 4.77	0.024	0.07
266392 Lachnospiraceae *Coprococcus*	10.33 ± 4.90	0.039	0.06
100100 Bacillaceae unclassified	−17.88 ± 6.19	0.005	0.11
128382 Veillonellaceae *Dialister*	−21.80 ± 5.72	<0.001	0.17
4319416 Bartonellaceae *Bartonella* unclassified	−27.37 ± 7.33	<0.001	0.17
100001 unclassified	−27.75 ± 11.19	0.016	0.08
1007180 Methylobacteriaceae *Methylobacterium*	−28.03 ± 13.61	0.043	0.06
1074945 Propionibacteriaceae *Propionibacterium granulosum*	−28.03 ± 13.61	0.043	0.06
2170756 Xanthomonadaceae *Stenotrophomonas geniculata*	−28.03 ± 13.61	0.043	0.06
579304 Lactobacillaceae	−28.03 ± 13.61	0.043	0.06
4473250 Paenibacillaceae *Aneurinibacillus migulanus*	−31.56 ± 12.59	0.015	0.08
Isovaleric acid			
1105591 Paraprevotellaceae *Paraprevotella*	37.88 ± 10.38	<0.001	0.16
1000148 Comamonadaceae unclassified	19.47 ± 9.45	0.043	0.06
839684 Lachnospiraceae unclassified	18.20 ± 7.28	0.015	0.08
100100 Bacillaceae unclassified	−20.32 ± 10.03	0.047	0.05
128382 Veillonellaceae *Dialister*	−28.92 ± 9.30	0.003	0.12
4319416 Bartonellaceae *Bartonella* unclassified	−34.65 ± 11.99	0.005	0.10

Operational taxonomic unit (OTU) number, family, and genus are shown; species are also shown when indicated. r^2^, square of Pearson’s correlation coefficient; SE, standard error.

## Data Availability

The data presented in this study are available in the Supplementary Materials.

## Supplementary Materials

The following are available online at https://www.mdpi.com/article/10.3390/microorganisms9122430/s1: Table S1: Plasma metabolomic data; Table S2: Fecal metabolomic data; Table S3: Fecal microbiota data; Table S4: Correlations of metabolomic and microbiota data.
